# Bioelectrical Impedance Analysis Results for Estimating Body Composition Are Associated with Glucose Metabolism Following Laparoscopic Sleeve Gastrectomy in Obese Japanese Patients

**DOI:** 10.3390/nu10101456

**Published:** 2018-10-08

**Authors:** Yoshinori Ozeki, Takayuki Masaki, Yuichi Yoshida, Mitsuhiro Okamoto, Manabu Anai, Koro Gotoh, Yuichi Endo, Masayuki Ohta, Masafumi Inomata, Hirotaka Shibata

**Affiliations:** 1 Department of Endocrinology, Metabolism, Rheumatology and Nephrology, Faculty of Medicine, Oita University, Yufu City, Oita 879-5593, Japan; ozeki23@oita-u.ac.jp (Y.O.); y-yoshida@oita-u.ac.jp (Y.Y.); mokamoto@oita-u.ac.jp (M.O.); manabua@oita-u.ac.jp (M.A.); gotokoro@oita-u.ac.jp (K.G.); hiro-405@oita-u.ac.jp (H.S.); 2 Department of Gastroenterological and Pediatric Surgery, Oita University, Yufu City, Oita 879-5593, Japan; endo@oita-u.ac.jp (Y.E.); ohta@oita-u.ac.jp (M.O.); inomata@oita-u.ac.jp (M.I.)

**Keywords:** obesity, sleeve gastrectomy, body composition, fat mass

## Abstract

We investigated the association between body composition and changes in glucose metabolism following laparoscopic sleeve gastrectomy (LSG) in obese Japanese patients. Thirty-two Class III obese patients were assessed before LSG and 3, 6, and 12 months postoperatively. Variables including fat mass (FM), % body fat (%FM), total and skeletal muscle mass (MM), the ratio of lower extremity MM to body weight (BW) (L/W), and the ratio of upper extremity MM to BW (U/W) were measured while using bioelectrical impedance analysis (BIA). LSG significantly decreased BW, FM, and %FM in all time periods observed after surgery with concomitant improvements in metabolic markers. MM was decreased at three months but maintained from 3–12 months post-surgery. Importantly, %MM, U/W, and the L/W ratio increased after LSG. Furthermore, change in FM was positively correlated with change in BW 12 months after LSG, whereas changes in %MM were negatively correlated with fasting plasma glucose (FPG) and hemoglobin A1c (HbA1c). Finally, multivariable stepwise regression analyses showed that changes in % total MM was an independent determinant of FPG and change in % skeletal MM was a significant independent determinant of HbA1c in Class III obese Japanese patients after LSG.

## 1. Introduction

Bariatric surgery is one of the most effective treatments for Class III obese patients. It significantly reduces obesity and lessens comorbid conditions, such as type-2 diabetes [1]. Among the surgical techniques utilized to promote weight loss, laparoscopic sleeve gastrectomy (LSG) is the most popular bariatric surgery and it is covered by health insurance in Japan [2,3]. LSG-associated body weight loss is also effective for improving type-2 diabetes [2,3].

Body composition assessments have an important role in clinical evaluation as well as in the monitoring of absolute and relative changes in fat mass (FM) and lean body mass during specific therapeutic regimens. Various assessment techniques have been evaluated in previous studies and different patterns have been reported [4,5,6]. Dual-energy X-ray absorptiometry (DXA) is traditionally used to assess body composition and it is considered to be valid and reliable [4]. However, the widespread application of this method is limited because it requires expensive equipment, trained technicians, and dedicated facilities. 

Bioelectrical impedance analysis (BIA) represents a simple and noninvasive means of assessing body composition [7,8,9]. The method is based on the principle that electrical current moves more freely through hydrated tissue and extracellular water than through adipose tissue, providing reproducible and rapidly obtained results [8,9]. However, obese patients exhibit variable levels of soft tissue hydration, which may lead to errors in the results of this method. In addition, obese individuals experience changes in body composition, which are characterized by an increased amount of FM [9].

The loss of muscle mass (MM) via conditions such as sarcopenia is involved in type 2 diabetes and obesity [10,11,12]. A combination of skeletal MM decline and excess adiposity is termed sarcopenic obesity, which increases the risks for disability and mortality [13]. Several indices of sarcopenia have also been defined, including the ratio of lower extremity muscle mass to body weight (L/W ratio) and the ratio of lower extremity muscle mass to upper extremity muscle mass [11,14]. Although several studies have analyzed body composition in obese subjects after bariatric surgery, little is known about the relationships between body compositions, particularly MM, and glucose metabolism disorders. The objective of the study was to examine the relationships between glucose metabolism and body composition, as measured by BIA, particularly MM. Therefore, we investigated the association between MM, including total and skeletal MM, L/W, U/W ratio, and glucose metabolism disorders in Class III obese Japanese patients.

## 2. Materials and Methods

### 2.1. Subjects and Protocol

We retrospectively recruited 50 consecutive Class III obese patients (mean body mass index (BMI) 45.1 ± 9.7 kg/m^2^, age 40.5 ± 9.1 years) undergoing LSG at Oita University Hospital from November 2013 to March 2018. Patients were selected for LSG according to the inclusion criteria standardized by the Ministry of Health, Labour, and Welfare of Japan. Eighteen subjects were excluded from the study between 0 months and 12 months after surgery. Inability to adhere to the testing schedule represented the key reason for exclusion. The final study cohort included 32 subjects, including 14 males and 18 females. In the present study, 10 of 17 patients with hypertension were taking hypertensive medication (Ca inhibitor, eight patients; ARB, nine patients; others, two patients). Three of 16 patients with hyperlipidemia were taking lipid-lowering medications (statins, one patient; fibrates, 0 patients; others, two patients). 18 patients did not have type-2 diabetes and eleven of 14 patients with type-2 diabetes were taking glucose-lowering medications (insulin, 0 patients; DPP-4 inhibitors, five patients; SGLT2 inhibitor, four patients; sulfonylureas, two patients; others, seven patients). None of the patients with type 2 diabetes was taking glucose-lowering medication to reduce glucose levels to the healthy range. In the present study, we examined time-course changes in obese patients with LSG; therefore, there was no control group (e.g., obese patients without LSG). Extensive clinical and hormonal endocrine evaluations were used to identify and exclude patients with endocrine diseases. The study design was in accordance with the Helsinki Declaration and was approved by the Ethical Committee of Oita University. The subjects gave their informed consent to participate in the study.

### 2.2. Biochemical Measurement and Blood Pressure

Blood was taken at 8:00 A.M. from the antecubital vein in patients in a recumbent position after an overnight fast. All of the patients underwent routine laboratory tests, including assays for plasma aspartate aminotransferase (AST), alanine transaminase (ALT), glutamic pyruvic transaminase (GTP), blood urea nitrogen (BUN), creatinine (Cr), low-density lipoprotein (LDL), triglycerides, high-density lipoprotein (HDL), glucose, and HbA1c. Fasting plasma glucose (FPG) was measured while using an enzymatic method (EIKEN, Tokyo, Japan). Hemoglobin A1c (HbA1c) was measured using high-performance liquid chromatography. BP was measured using the cuffoscillometric method between 8:00–10:00 A.M.

### 2.3. Anthropometry and Body Analyses

Body weight (BW), height, and BMI were measured for all subjects. BW was measured to the nearest 0.1 kg using digital scales; height was measured to the nearest 0.1 cm with the subjects wearing light indoor clothing. BMI was calculated from weight and height (kg/m^2^). Ideal body weights were calculated as the body weight at a BMI of 25. The percent of total body weight loss (%TBWL) after surgery was calculated as (BW loss/BW) × 100. Excess body weight loss was BW—ideal body weight and percent of excess body weight loss (%EBWL) after surgery was (BW loss/EBW) × 100.

### 2.4. Bioelectrical Impedance Analysis

Body composition was analyzed using a BIA device (In-Body 770 Biospace Co., Ltd., Tokyo, Japan). The method is based on the principle that lean body mass contains higher levels of water and electrolytes than fat tissue, and so these tissues can be distinguished based on electrical impedance. Segmental body composition was estimated using a patented eight-point tactile electrode system. The device uses six frequencies (1, 5, 50, 250, 500, and 1000 kHz) and produces 30 impedance values for five body segments: right and left upper extremities, trunk, and right and left lower extremities [15]. A previous validation study showed that both fat mass (FM) and fat free mass (FFM) evaluated by this device were highly correlated with measurements using dual-energy X-ray absorptiometry (correlation coefficient = 0.832 and 0.899, respectively) [16].

Body composition was determined according to the standard technique, with the subject in a standing position and the electrodes placed on the right and left upper hand and foot. Patients were evaluated after overnight fasting and with an empty bladder. They were asked to refrain from strenuous exercise during the one-day preceding the measurements. Weight and height were recorded, and a clinical examination was performed. Body composition was calculated from bioelectrical measurements and anthropometric data by applying the software that was provided by the manufacturer, which incorporated validated predictive equations for BW. FM, percent of fat mass (%FM), total MM, skeletal MM, bone mineral content, and total body fluid were assessed. %FM was calculated as the product of percent fat and weight. Lean mass was determined to be the difference between BW and fat weight. % total MM was calculated as total MM (kg)/weight (kg) × 100. % skeletal MM was calculated as skeletal MM (kg)/weight (kg) × 100. L/W was calculated as lower muscle per kg/BW. U/W was calculated as upper muscle per kg/BW, according to a previous study [11].

### 2.5. Statistical Analyses

The data are presented as means ± SDs and they were analyzed by commercial software (JMP13.2 SAS Institute, Cary, NC, USA). The data of time-course change for each parameter were statistically evaluated by one-way analysis of variance (ANOVA). In addition, the data of each time point were evaluated by a post-hoc multiple comparison. A *p* value of less than 0.05 was considered to be statistically significant. Simple (Spearman rank) correlation coefficients were calculated, and then multiple regression analyses were used to evaluate the independent associations of these variables. Multiple stepwise regression analyses were conducted to access the association between body composition and glycemic profiles after adjusting for potential confounders, including BW and body fluid. Body fluid was included as a covariate in the BW model, and BW and body fluid were included as covariates in the FPG and HbA1c models.

## 3. Results

### 3.1. Basal Clinical Characteristics of Obese Patients and Body Weight Changes after LSG

The descriptive characteristics of the subjects prior to surgery are shown in Table 1. BW and BMI were both decreased at 3, 6, and 12 months after LSG when compared to pre-surgery (*p* < 0.01 for each) (Table 1). There was no significant difference between 3 vs. 6, 6 vs. 12 or 3 vs. 12 months in BW and BMI. The levels of %TBWL and %EBWL at 6 and 12 months were both increased compared to them at 3 months (Table 1). At 12 months after LSG compared to pre-surgery, there was no significant difference between males and females in the change in BW (males −37.1 ± 14.1 kg vs. females −38.2 ± 10.6 kg: *p* = 0.80), FM (males −29.2 ± 15.5 kg vs. females −27.8 ± 12.1 kg: *p* = 0.52), and %FM (males −13.7 ± 8.2 kg vs. females −15.5 ± 7.8 kg: *p* = 0.64) after bariatric surgery. However, male and female patients significantly differed with regard to %TBW loss (male 27.9 ± 8.9% vs female −34.7 ± 7.0%: *p* = 0.02).

### 3.2. Changes in Fat Mass after LSG 

Observed FM and %FM were dramatically decreased at the 3, 6, and 12 month time points as compared to pre-surgery (*p* < 0.01 for each) (Table 1). In addition, both FM and %FM at 12 months were significantly decreased compared to them at three months (Table 1). Bone mineral content did not significantly change throughout the study (*p* > 0.1).

### 3.3. Muscle Mass Changes after LSG

Data on the MM, L/W, and U/W ratio over time are given in Table 1. The MM at 3, 6, and 12 months was decreased when compared to pre-surgery values, and MM was preserved 3 to 12 months after LSG. L/W and U/W ratio were significantly increased 3 or 6 months after LSG, and L/W ratio at 12 months increased compared to L/W at 3 months (*p* < 0.05). U/W ratio was unchanged from 3 month to 12 months after LSG. Observed total and skeletal %MM were dramatically increased at the 3, 6, and 12 month time points as compared to pre-surgery. In addition, both total and skeletal %MM at 12 months increased compared to %MM at three months. 

### 3.4. Time Course Changes in Plasma Metabolic Parameters

Levels of FPG, HbA1C, AST, ALT, GTP, and triglycerides were all significantly decreased after LSG at 3, 6, and 12 months compared to pre-surgery levels. Conversely, plasma HDL was increased after LSG at 6 and 12 months compared to pre-surgery levels. There was no significant difference between 3 vs. 6, 6 vs. 12, or 3 vs. 12 months in FPG, HbA1C, AST, ALT, GTP, and triglycerides (Table 1). Plasma LDL, BUN, and Cr did not significantly change throughout the time period.

### 3.5. Correlation between Changes in Body Compositions and Changes in Glycemic Metabolic Parameters 12 Months after LSG

**Figure 1:**
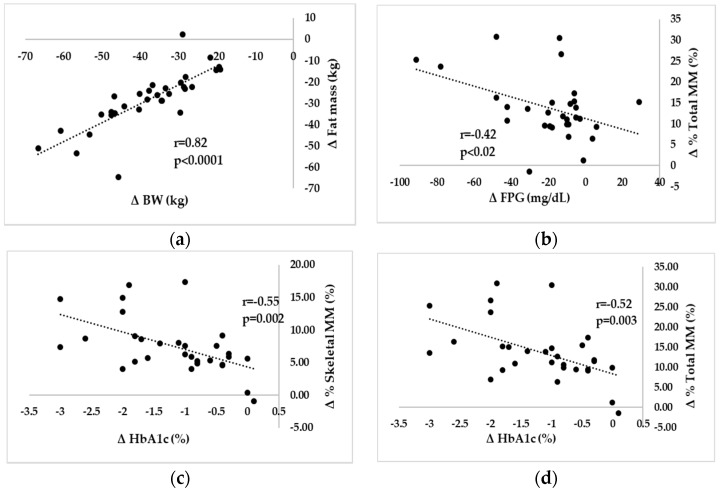
Correlation between changes in body compositions and changes in glycemic metabolic parameters. (**a**) relationship between BW and fat mass; (**b**) relationship between FPG and % total MM; (**c**) relationship between HbA1c and % skeletal MM; (**d**) relationship between HbA1c and % total MM, Variables: **Δ** (0–12 month) variables; BW: body weight, MM: muscle mass, FPG: fasting plasma glucose, HbA1c: hemoglobin A1c. Simple correlation coefficients were calculated. *r* = correlation coefficient. A *p* value of less than 0.05 was considered to be statistically significant.

Changes in BW were associated with changes in FM, %FM (*r* = 0.58; *p* = 0.001), total MM (*r* = 0.43; *p* = 0.02), skeletal MM (*r* = 0.46; *p* = 0.01), % total MM (*r* = −0.57; *p* = 0.001), % skeletal MM (*r* = −0.55; *p* = 0.001), U/W (*r* = −0.52; *p* = 0.003), and L/W (*r* = −0.64; *p* < 0.001). Changes in FPG were correlated with changes in %FM (*r* = 0.42; *p* = 0.02), %total MM, %skeletal MM (*r* = −0.40; *p* = 0.03), and L/W (*r* = −0.38; *p* = 0.04). Changes in HbA1c were correlated with changes in %FM (*r* = 0.52; *p* = 0.003), %MM, U/W (*r* = −0.43; *p* = 0.02), and L/W (*r* = −0.41; *p* = 0.02). Figure 1 shows the data on correlations between ΔBW-ΔFM, ΔFPG-Δ%total MM, and ΔHbA1c-Δ%MM.

### 3.6. Relationships among Changes in Body Weight, FPG, and HbA1c

Multiple stepwise regression analyses were conducted to assess the association between body composition and glycemic profiles after adjusting for potential confounders, including BW and body fluid (Table 2). Multiple stepwise regression analyses were carried out for changes in BW as a dependent variable, including changes in FM, %FM, MM, %MM, U/W, and L/W as independent variables. Only changes in FM were independently correlated with changes in BW.

To examine the contribution of changes in %FM, %MM, and L/W to changes in FPG, multivariate stepwise regression analyses were used. When changes in BW and changes in body fluid entered into the equation as covariates, only changes in %total MM remained as an independent determinant of changes in FPG. In addition, multiple stepwise regression analyses were carried out for changes in HbA1c as a dependent variable, including changes in %FM, %MM, L/W, U/W, body fluid, BW, and HbA1c as independent variables. Only changes in % skeletal MM were independently correlated with changes in HbA1c.

## 4. Discussion

The present study demonstrated that BW, FM, and %FM decreased, while % total MM and % skeletal MM increased after LSG. In addition, changes in FM and %MM were correlated with obesity, FPG, and HbA1c in Class III obese Japanese patients after LSG.

Bariatric surgery is used to treat obesity and comorbid conditions. In terms of weight loss, a sustained reduction of ≥50% excess weight is considered as a success. Recent meta-analyses and systematic reviews have reported a %EBWL close to 60% one year after a gastric bypass with ranges from 33% to 77% [17,18,19]. In the present study, LSG reduced BW in all time periods observed. %TBWL was 31.7 ± 8.5% and %EBWL was 79.6 ± 28.9% at 12 months compared to pre-surgery weights. The results are similar to previous studies [17,18,19]. In addition, several studies described the data in Japanese patients following bariatric surgery [20,21]. Japan nationwide surveys of bariatric surgery demonstrated the mean age of obese patients was 41 years, and mean BMI was 42 kg/m^2^ [20]. After LSG with duodeno-jejunal bypass, %TBWL was 29% at one year in Japan [21]. The age and changes in weight are also almost comparable to data in the present study.

The great weight loss observed in our patients prompted concern regarding the impact of weight loss on body composition, including FM and MM. The excess weight in obese patients is mainly due to FM [19,22], and several studies have demonstrated a close correlation between the content of body fat and several cardiovascular and metabolic diseases. In our study, there was a significant loss of FM in all patients. One year after surgery, the %FM reached levels within 30%, a significant finding given that FM loss is important to improve glucose metabolism disorders.

Contrary to FM loss, a reduction of MM has undesirable effects on diabetic obese patients. Our data demonstrate the time course of several changes to muscle composition in obese Japanese patients. This study showed that MM are maintained in patients from 3 to 12 months after LSG. L/W ratio was increased three months after LSG, and L/W ratio also increased at 12 months compared to three months. Total and skeletal %MM were dramatically increased at the 3, 6, and 12 month time points as compared to pre-surgery. In addition, %MM was increased at 12 months compared to three months after LSG. This resulted in a positive change in body composition after LSG in obese patients.

There is question about the utility of the BIA method for examining body composition of Class III obese patients. Several studies have validated the accuracy of body composition determined by BIA in bariatric surgery patients. Parallel measurements of body composition using BIA and DXA in a homogeneous normo-hydrated group of obese subjects post-LAGB shows high agreement between the results obtained by BIA and DXA. These findings indicate that the BIA method might be useful as an alternative to DXA [16,23]. To avoid the influence of body fluids on MM, body compositions were adjusted for body fluid in the present study.

We also examined sex-specific changes in body composition after LSG. It is well recognized that women have more body fat than men at the same relative BMI. A previous study observed that obese female patients had higher body fat and lower muscle percentages than males [24]. Additionally, several studies have tested for gender differences in weight and fat loss after bariatric surgery [25,26]. One study found no significant differences in the BMIs of males and females after bariatric surgery, although male patients continued to weigh significantly more than females and lost significantly more pounds than did females after bariatric surgery [25]. Another study found that %EBWL differed significantly between male and female patients after LSG [26]. In the present study, changes in BW, FM, and %FM did not differ between the sexes. However, %BW loss differed significantly between male and female patients. This may be due to the difference between the absolute value and the % of BW. In this study, however, changes in BMI, body fat, and muscle weight percentage did not differ between the sexes. The results are difficult to explain. It appears that changes in individual body composition with weight loss are highly variable even within the same sex. Several studies have demonstrated a mechanistic link between menstruation and body composition, but it is clear that additional studies are needed to further clarify the potential sex differences in body composition measures after bariatric surgery.

A recent study described the effect of bariatric surgery on diabetes outcomes persists fifth year after surgery in non-Japanese individuals [27]. In the present study, we examined the relationships between changes in body composition and plasma glycemic parameters in Japanese patients for 12 months following LSG. FPG and HbA1c were dramatically improved after LSG. Changes in FM were correlated with changes in BW after LSG. In addition, changes in %MM were negatively correlated with changes in FPG and HbA1c up to 12 months after LSG. Multivariable regression analyses showed that changes in FM were independent and significant determinants of BW loss, changes in % total MM were significant independent determinants of FPG, and changes in % skeletal MM were significant independent determinants of HbA1c. The precise significance of differences between changes in %total MM and changes in % skeletal MM are unknown, however, our results show that changes in %MM are significant determinants of FPG and HbA1c in obese Japanese patients after LSG. In fact, enhanced skeletal muscle offers great benefits for glucose regulation via muscle-intrinsic mechanisms. Also, enhanced muscle growth has positive effects on fat metabolism and insulin resistance/sensitivity through several molecular mechanisms, such as increasing adiponectin expression [28].

Limitations to our study included statistical overfitting because of a small sample size and potential confounding factors. BIA is sensitive to hydration, and we did not measure hydration status or caffeine intake in this study. The study design did not allow us to examine a causal relationship, and future prospective studies including appropriate control patients would help to clarify some of the outcomes. Further studies are necessary to clarify the physiological mechanisms that contribute to changes in body composition. In addition, although almost all patients were medication-free after LSG, we cannot fully exclude a possible influence of several temporal medications. Finally, more detailed and longer studies are needed to validate the effects of LSG on muscle parameters, including muscle strength and quality.

## Figures and Tables

**Table 1:** Time-course changes in body weight, plasma metabolic parameters, and body composition.

	pre-LSG	Three Months	Six Months	Twelve Months	f	*F*-Value	*p*-Value
Body weight (kg)	120.0 ± 25.9	91.9 ± 21.3 ***	85.7 ± 20.8 ***	82.3 ± 22.2 ***	3	18.4	<0.001
Total body weight loss (kg)		28.1 ± 8.7	34.3 ± 10.0 ^†^	37.8 ± 12.1 *^†††^	2	7.16	0.001
%TBWL		23.4 ± 5.5	28.6 ± 6.0 ^†^	31.7 ± 8.5 *^†††^	2	12.3	<0.001
%EBWL		51.9 ± 21.4	71.9 ± 24.3 ^†^	79.6 ± 28.9 *^††^	2	5.51	0.006
BMI (kg/m^2^)	45.1 ± 9.7	34.6 ± 7.9 ***	32.2 ± 7.7 ***	30.8 ± 8.1 ***	3	19.1	<0.001
Systolic blood pressure (mmHg)	136.1 ± 19.6	123.1 ± 18.2 **	121.4 ± 20.0 **	121.2 ± 16.5 **	3	4.74	0.004
Diastolic blood pressure (mmHg)	84.8 ± 14.5	79.8 ± 12.4	77.4 ± 12.3	77.1 ± 12.3	3	2.46	0.07
Fasting plasma glucose (mg/dL)	109.6 ± 21.0	91.8 ± 15.7 ***	89.1 ± 9.9 ***	91.1 ± 15.8 ***	3	10.8	<0.001
HbA1c (%)	6.4 ± 0.9	5.3 ± 0.4 ***	5.3 ± 0.3 ***	5.3 ± 0.3 ***	3	31.2	<0.001
Triglycerides (mg/dL)	163.9 ± 92.0	98.8 ± 33.8 ***	92.0 ± 30.6 ***	83.8 ± 39.7 ***	3	13.5	<0.001
HDL cholesterol (mg/dL)	45.7 ± 8.7	47.1 ± 9.6	53.8 ± 11.4 **^†^	59.1 ± 14.1 ***^†††^	3	9.45	<0.001
LDL cholesterol (mg/dL)	125.2 ± 28.7	122.9 ± 32.4	125.8 ± 28.0	122.9 ± 35.4	3	0.07	0.97
BUN (mg/dL)	12.5 ± 3.0	11.3 ± 4.0	12.3 ± 3.7	13.6 ± 3.7	3	2.32	0.08
Creatinine (mg/dL)	0.70 ± 0.21	0.69 ± 0.17	0.71 ± 0.15	0.72 ± 0.17	3	0.21	0.89
AST (IU/L)	37.7 ± 24.9	19.0 ± 5.7 ***	15.8 ± 3.2 ***	16.0 ± 4.2 ***	3	20.7	<0.001
ALT (IU/L)	55.4 ± 45.1	18.4 ± 10.2 ***	12.8 ± 4.1 ***	12.9 ± 5.5 ***	3	24.7	<0.001
GTP (IU/L)	45.3 ± 34.9	20.1 ± 16.4 ***	17.7 ± 17.2 ***	16.1 ± 10.1 ***	3	12.9	<0.001
FM (%)	48.1 ± 6.9	39.9 ± 10.2 ***	35.9 ± 9.8 ***	33.4 ± 10.5 ***^††^	3	14.8	< 0.001
FM (kg)	56.5 ± 19.0	37.3 ± 16.3 ***	31.2 ± 14.8 ***	28.1 ± 15.3 ***^†^	3	19.7	<0.001
Total MM (kg)	56.7 ± 11.6	50.6 ± 10.3 *	50.6 ± 11.0 *	50.0 ± 10.7 *	3	2.67	0.05
Total MM/BW	0.49 ± 0.06	0.57 ± 0.10 **	0.61 ± 0.10 ***	0.63 ± 0.10 ***^††^	3	14.5	<0.001
Skeletal MM (kg)	33.4 ± 7.2	29.3 ± 6.4 *	29.2 ± 6.7 *	29.0 ± 6.7 **	3	3.18	0.03
Skeletal MM/BW	0.29 ± 0.04	0.33 ± 0.06 *	0.35 ± 0.06 ***	0.36 ± 0.06 ***^†^	3	10.9	<0.001
Upper Skeletal MM/BW	0.06 ± 0.01	0.07 ± 0.01	0.07 ± 0.01 **	0.07 ± 0.01 **	3	4.17	0.008
Lower Skeletal MM/BW	0.16 ± 0.02	0.19 ± 0.03 ***	0.20 ± 0.03 ***	0.21 ± 0.03 ***^†^	3	15.3	<0.001

LSG: laparoscopic sleeve gastrectomy; TBWL: total body weight loss; EBWL: excessive body weight loss; BMI: body mass index; BUN: blood urea nitrogen; HDL: high-density lipoprotein; LDL: low-density lipoprotein; AST: aspartate aminotransferase; ALT: alanine transaminase; GTP: glutamic pyruvic transaminase; HbA1c: hemoglobin A1c; FM: fat mass; MM: muscle mass; BW: body weight. f, degrees of freedom; *F*-value and *p*-value for each parameter are described in right columns (indicate significant time-course changes assessed by ANOVA analysis). * *p* < 0.05, ** *p* < 0.01, *** *p* < 0.001 vs. pre-surgery; ^†^ *p* < 0.05, ^††^ *p* < 0.01, ^†††^ *p* < 0.001 vs. 3 month (indicate significant changes between each time assessed by posthoc multiple comparison).

**Table 2:** Multiple stepwise regression models with **Δ**BW, **Δ**FPG, and **Δ**HbA1c as dependent valuables.

Model	Independent Variables	*F* Value	*p* Value
BW	Fat mass (kg)	59.6	<0.001 **
FPG	% Total MM (%)	5.9	0.022 *
HbA1c	% Skeletal MM (%)	11.2	0.002 **

Independent variables: **Δ** (0–12 month) variables, BW: body weight, FPG: fasting plasma glucose; HbA1c: hemoglobin A1c, MM: muscle mass, **Δ** (0–12 month), The covariates in BW model is body; fluid and covariates in FPG and HbA1c model are BW and body fluid. * *p* < 0.05, ** *p* < 0.01.

## Abbreviations

LSG	laparoscopic sleeve gastrectomy
BW	Body weight
BIA	bioelectrical impedance analysis
DX	Dual-energy X-ray absorptiometry
FM	fat mass
MM	muscle mass weight
%MM	% muscle mass
L/W	the ratio of lower extremity MM to BW
U/W	the ratio of upper extremity MM to BW
TBWL	total body weight loss
EBWL	excessive body weight loss
AST	aspartate aminotransferase
ALT	alanine transaminase
GTP	glutamic pyruvic transaminase
BUN	blood urea nitrogen
Cr	creatinine
LDL	low-density lipoprotein
HDL	high-density lipoprotein
FPG	fasting plasma glucose
HbA1c	Hemoglobin A1c

## References

[1] Shoar S., Mahmoudzadeh H., Naderan M. (2017). Long-term outcome of bariatric surgery in morbidly obese adolescents: A systematic review and meta-analysis of 950 patients with a minimum of 3 years follow-up. Obes. Surg..

[2] Wang Y., Yi X., Li Q. (2016). The effectiveness and safety of sleeve gastrectomy in the obese elderly patients: A systematic review and meta-analysis. Obes. Surg..

[3] Switzer N.J., Prasad S., Debru E. (2016). Sleeve gastrectomy and type 2 diabetes mellitus: A systematic review of long-term outcomes. Obes. Surg..

[4] Ramírez-Vélez R., Correa-Bautista J.E., González-Ruíz K. (2016). Predictive validity of the body adiposity index in overweight and obese adults using dual-energy x-ray absorptiometry. Nutrients.

[5] Gómez-Ambrosi J., González-Crespo I., Catalán V. (2018). Clinical usefulness of abdominal bioimpedance (ViScan) in the determination of visceral fat and its application in the diagnosis and management of obesity and its comorbidities. Clin. Nutr..

[6] Umemura A., Sasaki A., Nitta H. (2014). Effects of changes in adipocyte hormones and visceral adipose tissue and the reduction of obesity-related comorbidities after laparoscopic sleeve gastrectomy in Japanese patients with severe obesity. Endocr. J..

[7] Widen E.M., Strain G., King W.C. (2014). Validity of bioelectrical impedance analysis for measuring changes in body water and percent fat after bariatric surgery. Obes. Surg..

[8] Buffa R., Mereu E., Comandini O. (2014). Bioelectrical impedance vector analysis (BIVA) for the assessment of two-compartment body composition. Eur. J. Clin. Nutr..

[9] Xiao J., Purcell S.A., Prado C.M. (2017). Fat mass to fat-free mass ratio reference values from NHANES III using bioelectrical impedance analysis. Clin. Nutr..

[10] Tanaka K.I., Kanazawa I., Sugimoto T. (2016). Reduced muscle mass and accumulation of visceral fat are independently associated with increased arterial stiffness in postmenopausal women with type 2 diabetes mellitus. Diabetes Res. Clin. Pract..

[11] Hamasaki H., Kawashima Y., Adachi H. (2015). Associations between lower extremity muscle mass and metabolic parameters related to obesity in Obese Japanese patients with type 2 diabetes. Peer. J..

[12] Wannamethee S.G., Atkins J.L. (2015). Muscle loss and obesity: The health implications of sarcopenia and sarcopenic obesity. Proc. Nutr. Soc..

[13] Rossi A.P., Bianchi L., Volpato S. (2017). Dynapenic abdominal obesity as a predictor of worsening disability, hospitalization, and mortality in older adults: Results from the In CHIANTI study. J. Gerontol. Biol. Sci. Med. Sci..

[14] Dixon J.B., Bhasker A.G., Lambert G.W. (2016). Leg to leg bioelectrical impedance analysis of percentage fat mass in obese patients: Can it tell us more than we already know?. Surg. Obes. Relat. Dis..

[15] Anderson L.J., Erceg D.N., Schroeder E.T. (2012). Utility of multifrequency bioelectrical impedance compared with dual-energy X-ray absorptiometry for assessment of total and regional body composition varies between men and women. Nutr. Res..

[16] Faria S.L., Faria O.P., Cardeal M.D. (2014). Validation study of multi-frequency bioelectrical impedance with dual-energy X-ray absorptiometry among obese patients. Obes. Surg..

[17] Kim H.J., Madan A., Fenton-Lee D. (2014). Does patient compliance with follow-up influence weight loss after gastric bypass surgery? A systematic review and meta-analysis. Obes. Surg..

[18] Zhang C., Yuan Y., Qiu C. (2014). A meta-analysis of 2-year effect after surgery: Laparoscopic Roux-en-Y gastric bypass versus laparoscopic sleeve gastrectomy for morbid obesity and diabetes mellitus. Obes. Surg..

[19] Qi L., Guo Y., Liu C.Q. (2017). Effects of bariatric surgery on glycemic and lipid metabolism, surgical complication and quality of life in adolescents with obesity: A systematic review and meta-analysis. Surg. Obes. Relat. Dis..

[20] Haruta H., Kasama K., Ohta M. (2017). Long-Term Outcomes of Bariatric and Metabolic Surgery in Japan: Results of a Multi-Institutional Survey. Obes. Surg..

[21] Seki Y., Kasama K., Haruta H. (2017). Five-Year-Results of Laparoscopic Sleeve Gastrectomy with Duodenojejunal Bypass for Weight Loss and Type 2 Diabetes Mellitus. Obes. Surg..

[22] Rayner J.J., Banerjee R., Francis J.M. (2015). Normalization of visceral fat and complete reversal of cardiovascular remodeling accompany gastric bypass, not banding. J. Am. Coll. Cardiol..

[23] Tewari N., Awad S., Macdonald I.A. (2018). A comparison of three methods to assess body composition. Nutrition.

[24] Van Caenegem E., Wierckx K., Taes Y. (2012). Bone mass, bone geometry, and body composition in female-to-male transsexual persons after long-term cross-sex hormonal therapy. J. Clin. Endocrinol. Metab..

[25] Tymitz K., Kerlakian G., Engel A. (2007). Gender differences in early outcomes following hand-assisted laparoscopic Roux-en-Y gastric bypass surgery: Gender differences in bariatric surgery. Obes. Surg..

[26] Perrone F., Bianciardi E., Benavoli D. (2016). Gender Influence on Long-Term Weight Loss and Comorbidities after Laparoscopic Sleeve Gastrectomy and Roux-en-Y Gastric Bypass: A Prospective Study with a 5-Year Follow-up. Obes. Surg..

[27] Ikramuddin S., Korner J., Lee W.J. (2018). Lifestyle Intervention and Medical Management with vs without Roux-en-Y Gastric Bypass and Control of Hemoglobin A1c, LDL Cholesterol, and Systolic Blood Pressure at 5 Years in the Diabetes Surgery Study. JAMA.

[28] Yang J. (2014). Enhanced skeletal muscle for effective glucose homeostasis. Prog. Mol. Biol. Transl. Sci..

